# The Dishonest Druggist

**Published:** 1905-02

**Authors:** 


### THE DISHONEST DRUGGIST.

Dishonesty of any sort is to be condemned, but there is no language strong enough, no penalty severe enough adequately to denounce and to punish that lowest form of dishonesty by which a man seeks to profit by endangering health and even life itself.

The dishonest maker or retailer of food products may do more or less serious injury to the health of those who buy his impure goods; but the dishonest dispenser of medicine may be directly responsible for the lives of the sick who have depended on him to give them the pure remedies prescribed by their physicians.

There is nothing much lower in the scale of mendacity than the druggist who knowingly sells impure drugs or dispenses inferior substances or substitutes for the ingredients set down in a physician's prescription. In this class also is the druggist who sends to the patient a prescribed remedy deficient in some of its ingredients. Yet what does the record of a recent investigation in this city show?

Prescriptions were sent to 139 druggists, each one calling for pure aristol. Of the dispensed articles twenty-three showed no trace of aristol, sixty-six had 80 per cent. of impurity, ten had 20 per cent. and nine had 10 per cent. Only thirty-one were found to be pure and as prescribed.

Only two conclusions may be drawn from these tests—out of 139 druggists who daily, in dispensing, take human life into their hands, 108 are either deliberately dishonest or so careless or incompetent that they are unfit to be intrusted with the dispensing of medicines. Of course no one will question the unfitness of the dishonest ones.

In this investigation, the results of which have been placed in the hands of the State Board of Pharmacy, it was found that many druggists had bought a "cheap" aristol which chemical tests proved to be an inert substance known as "Fuller's earth." Other brands of the chemical contained a small percentage of aristol and a very large percentage of impurity. It is the business of the druggist to know whether or not he is buying pure drugs. If he does not know how to tell this, or does not take pains to find out, then he should not be allowed to dispense.

The dishonest, the careless, or the incompetent druggist, is a menace to every one. No person can tell when he may be sick, when he may need a physician, when he may have to send a prescription to a druggist. The Board of Pharmacy owes it to the public to prosecute every dishonest druggist, every incompetent or careless dispenser, to let it be known that such men are unsafe, so that persons who have to buy medicines may give them a wide berth.—*Chicago Evening Post, Dec. 7, 1904.*

